# Coronary calcium density in relation to coronary heart disease and cardiovascular disease in adults with diabetes or metabolic syndrome: the Multi-ethnic Study of Atherosclerosis (MESA)

**DOI:** 10.1186/s12872-022-02956-4

**Published:** 2022-12-09

**Authors:** Yanglu Zhao, Shaista Malik, Michael H. Criqui, Matthew A. Allison, Matthew J. Budoff, Veit Sandfort, Nathan D. Wong

**Affiliations:** 1 Department of Epidemiology, University of California Los Angeles, Los Angeles, USA; 2 Heart Disease Prevention Program, University of California Irvine, Irvine, USA; 3 Division of Preventive Medicine, University of California San Diego, San Diego, USA; 4 Lindquist Institute, Harbor-UCLA Medical Center, Los Angeles, USA; 5 Radiology & Imaging Sciences, National Institutes of Health, Bethesda, USA

**Keywords:** Coronary calcium density, Coronary heart disease, Cardiovascular disease, Diabetes mellitus, Metabolic syndrome

## Abstract

**Background:**

Coronary artery calcium (CAC) density is inversely associated with coronary heart disease (CHD) and cardiovascular disease (CVD) risk. We examined this relation in those with diabetes mellitus (DM) or metabolic syndrome (MetS).

**Methods:**

We studied 3,818 participants with non-zero CAC scores from the Multiethnic Study of Atherosclerosis and classified them as DM, MetS (without DM) or neither DM/MetS. Risk factor-adjusted CAC density was calculated and examined in relation to incident CHD and CVD events over a median follow-up of 15 years among these three disease groups.

**Results:**

Adjusted CAC density was 2.54, 2.61 and 2.69 among those with DM, MetS or neither DM/MetS. Hazard ratios (HRs) for CHD per 1 SD increase of CAC density was 0.91 (95% CI: 0.72–1.16), 0.70 (95% CI: 0.56–0.87) and 0.79 (95% CI: 0.66–0.95) for those with DM, MetS or neither DM/MetS groups and were 0.77 (95% CI: 0.64–0.94), 0.83 (95% CI: 0.70–0.99) and 0.82 (95% CI: 0.71–0.95) for CVD, respectively. Adjustment for CAC density increased the HRs of CAC volume for CHD/CVD events. Compared to prediction models with or without single CAC measures, c-statistics of models with CAC volume and density were the highest ranging 0.67–0.72.

**Conclusion:**

CAC density is lower among patients with DM or MetS than those with neither DM/MetS and is inversely associated with future CHD/CVD risk among them. Including CAC density in risk assessment among those with MetS may improve prediction of CHD and CVD.

**Supplementary Information:**

The online version contains supplementary material available at 10.1186/s12872-022-02956-4.

## Background

Higher coronary artery calcium (CAC) scores have been shown to be independently related to higher coronary heart disease (CHD) as well as cardiovascular disease (CVD) risk in multiple studies [1–3]. Recent studies showed that other measures of calcified plaque, including CAC composition, location and distributional pattern were also related to future CVD risk [4–8]. Notably, Criqui et al. described the quantification of density of the total calcified plaque based on Agatston score and found CAC density was inversely related to future CHD and CVD risk and improved event risk prediction [9]. The study implied that when the CAC volume was comparable, denser calcified plaques were more stable and less likely to cause clinical events than non-calcified plaques [10, 11].

Not known is whether CAC density is related to other cardiometabolic conditions, especially diabetes mellitus (DM) or metabolic syndrome (MetS). In addition, it is not established whether the inverse association of CAC density with CVD risk differs in patients with DM or MetS. Given that patients with MetS and DM often have more pro-inflammatory, pro-oxidant and pro-thrombotic stimuli as well as higher statin usage that could potentially modify the coronary plaque composition, it is questionable whether the density of plaque plays the same protective role for CVD compared to metabolically healthy individuals [12]. We aimed to examine the CAC density in association with MetS and DM, as well as the relationship of CAC density with long-term CHD and CVD event risk among patients with these conditions.

## Methods

### Sample selection

Multiethnic Study of Atherosclerosis (MESA) is a population-based, prospective cohort study involving 6814 persons aged 45–84 years old free of clinical CVD at baseline. Full details of the MESA design have been previously published [13]. Participants were recruited during 2000 and 2002 in six US field centers from four race/ethnic groups of Caucasian (38%), African American (28%), Hispanic American (22%), or Chinese American (12%). All participants gave written informed consent. The original MESA study was approved by the institutional review boards at each field center.

Participants with at least one valid non-zero Agatston CAC score in Exam 1, 2 or 3 and follow-up for CHD events and CVD events were included in our study. Subjects were excluded if they had a missing CAC score, or score of zero where CAC density cannot be calculated, or if they had an incomplete risk factor profile [age, sex, race, blood pressure, body mass index (BMI), waist circumference, high-density lipoprotein-cholesterol (HDL-C), triglycerides, total cholesterol, glucose level, smoking status, education, hypertension medication, diabetes medication or lipid-lowering medication], or had incident events prior to their initial non-zero CAC score as used for the study baseline measure (Additional file 1: Fig. S1).

DM was defined as diagnosed DM, having a calibrated fasting serum glucose ≥ 7.0 mmol/L [126 mg/dL], or taking hypoglycemia medication or insulin, any of which were present at the time of or before their first valid non-zero Agatston score was reported. MetS was defined as having at least three of the following five conditions at the time of or before their first valid non-zero Agatston score: (1) waist circumstance ≥ 102 cm (male) or 88 cm (female); (2) triglycerides ≥ 1.8 mmol/L [150 mg/dL]; (3) HDL-C < 1.0 mmol/L [40 mg/dL] for male or < 1.3 mmol/L [50 mg/dL] for female; (4) blood pressure ≥ 130/85 mmHg or using hypertension medication; (5) fasting glucose ≥ 5.6 nmol/L [100 mg/dL] or using medication for hyperglycemia. Those who were defined as DM, regardless of the presence of MetS, were included in the DM group.

### CAC measures and risk factor assessment

The methodology for acquisition and interpretation of the CAC scans has been reported previously [14]. CAC area score and CAC density for each participant were calculated with the method introduced by Criqui et al. [9] The CAC area score (mm^2^) was obtained from dividing total CAC volume (mm^3^) by slice thickness (3 mm for electron-beam CT in three study centers and 2.5 mm for multidetector CT in other three sites). The CAC density was calculated from the Agatston score divided by CAC area score. For instance, a subject had a Agatston score of 24.92 and CAC volume score of 37.38 mm^3^ from 2.5 mm-thick slice multidetector CT. The area score was 37.38/2.5 = 14.95 mm^2^ and the CAC density was 24.92/14.95 = 1.67. Since previous analyses in MESA have shown log linear relationships between CAC and CVD risk, the Agatston score and CAC volume were log transformed as ln(score + 1) [15].

Information on demographics, smoking status, medical conditions, family history etc. was obtained by questionnaire. A central laboratory (University of Vermont, Burlington, VT, USA) measured concentrations of total and HDL-C, triglycerides and plasma glucose. Resting blood pressure was measured three times with the participant in the seated position. The average of the last two blood pressures was used. 10-year atherosclerotic cardiovascular disease (ASCVD) risk was calculated from age, sex, race/ethnicity, smoking, DM, total cholesterol, HDL-C, systolic blood pressure and hypertension medication [16].

### Follow-up and endpoint ascertainment

The cohort was followed from the date of the first non-zero Agatston score report in exam 1, 2 or 3 through the end of year 2017 with mean ± SD follow-up of 12.8 ± 4.5 years (range: 0.2–17.4 years). At intervals of 9–12 months, a telephone interviewer inquired about interim hospital admissions, cardiovascular diagnoses, and deaths. MESA obtained medical records for about 99% of hospitalized events and information about 97% of outpatient cardiovascular diagnoses. We followed the participants for all CHD events as our primary endpoint and all CVD events as our secondary endpoint. All CHD endpoints included fatal and non-fatal myocardial infarction, resuscitated cardiac arrest, probable angina followed by revascularization, definite angina, cardiac revascularization or CHD death. All CVD endpoints included above CHD events, fatal/nonfatal stroke, heart failure (HF) and CVD death. Myocardial infarction (MI) and CHD death were also examined as additional endpoints.

### Statistical analysis

#### Association of CAC density and CAC volume and baseline DM or MetS status

We examined the distribution of CAC score, CAC volume, and CAC density as well as other baseline characteristics in those with MetS, DM, or neither DM/MetS. Continuous variables were compared by ANOVA and categorical variables were compared by the Chi-square test of proportions. Adjusted CAC density and log transformed CAC volume [ln(Volume)] were compared among those with and without MetS and DM using ANCOVA adjusted for ln(Volume) or CAC density, age, sex, race/ethnicity, education, smoking status, statin use and hypertension medication.

#### CAC density and CAC volume in association with CHD/CVD in DM/MetS subgroups

The Cox proportional hazards regression model was used to examine ln(Volume) and CAC density with relation of incident CHD/CVD events after adjustment of 10-year ASCVD risk, race/ethnicity, education, BMI and statin therapy. CAC volume was adjusted in the model when we examined CAC density in association with the outcomes; CAC density was adjusted in the model when we examined CAC volume in association with the outcomes. Interaction tests of CAC density with DM and MetS subgroups were included in the Cox models to test for effect modification. In sensitivity analysis, models were further adjusted for high-sensitive C reactive protein (hs-CRP), interleukin-6 (IL-6) and fibrinogen. Spline Cox PH models were used to determine if there is a presence of a cut point below which there is a significant increase in CHD/CVD risk.

#### Predictive value of CAC density and CAC volume scores in CHD/CVD risk assessment

Harrell’s c-statistics were used to examine the added predictive value of CAC density for future CHD/CVD events. We set Model 1 (base model) as 10-year ASCVD risk + race/ethnicity + BMI + statin therapy, Model 2 as base model + ln(CAC score), Model 3 as base model + ln(Volume) and Model 4 as base model + ln(Volume) + CAC density. Harrell’s c-statistics of Model 4 were compared with other three models.

Analyses were performed using SAS version 9.4. A *p* value < 0.05 (and a *p* value < 0.1 for interaction test) by the two-tailed test was considered statistically significant.

## Results

### Association of CAC density and CAC volume and DM or MetS status

In total, 3,818 MESA participants (56.3% male) were included in our study with a mean age of 66.5 (± 9.0) years old. Among them 668 (17.5%) subjects had DM, 1,122 (29.4%) had MetS without DM and 2,028 (53.1%) had neither DM/MetS. Comparisons of all clinical measures were significantly different among the three groups and were more unfavorable in those with DM or MetS compared to those with neither DM/MetS group (Table 1). Agatston scores and CAC volumes were highest in those with DM and lowest in those with neither DM/MetS, while the difference in CAC density was relatively small, with the values being highest in those with neither DM/MetS (mean ± SD density: 2.60 ± 0.77) and the lowest in those with MetS (mean ± SD density: 2.53 ± 0.75).

**Table 1:** Baseline Characteristics

	DM (n = 668)	MetS (n = 1122)	Neither DM/MetS (n = 2028)	*p* value
Age, years	66.4 ± 9.0	65.7 ± 9.5	66.0 ± 9.7	0.14
Male	389 (58.2%)	543 (48.4%)	1217 (60.0%)	< 0.01
*Race/ethnicity*				
Caucasian	157 (23.5%)	499 (44.5%)	991 (48.9%)	< 0.01
African American	239 (35.8%)	251 (22.4%)	467 (23.0%)	< 0.01
Chinese	81 (12.1%)	108 (9.6%)	251 (12.4%)	0.06
Hispanic	191 (28.6%)	264 (23.5%)	319 (15.7%)	< 0.01
Current smokers	81 (12.1%)	135 (12.0%)	267 (13.2%)	0.59
BMI, kg/m^2^	30.5 ± 6.0	30.7 ± 5.0	26.6 ± 4.6%	< 0.01
SBP, mmHg	134.0 ± 22.8	134.8 ± 20.9	126.2 ± 21.0	< 0.01
Cholesterol, mmol/L [mg/dL]	4.9 ± 1.1 [188.8 ± 40.7]	5.1 ± 1.0 [195.6 ± 38.1]	5.1 ± 0.9 [195.4 ± 34.6]	0.0001
HDL-C, mmol/L [mg/dL]	1.2 ± 0.3 [45.9 ± 13.1]	1.1 ± 0.3 [42.8 ± 10.5]	1.4 ± 0.4 [54.6 ± 14.8]	< 0.01
10-year ASCVD score, %	30.5 ± 18.0	17.0 ± 12.2	15.0 ± 11.9	< 0.01
Hypertension treatment	435 (65.1%)	645 (57.5%)	678 (33.4%)	< 0.01
Statin treatment	217 (32.5%)	254 (22.6%)	376 (18.5%)	< 0.01
CAC Agatston score	345.9 ± 645.3	241.1 ± 494.4	237.4 ± 485.4	< 0.01
CAC volume score, mm^3^	307.8 ± 545.2	213.9 ± 410.4	208.5 ± 402.1	< 0.01
CAC density	2.59 ± 0.76	2.53 ± 0.75	2.60 ± 0.76	0.03

Continuous variables were presented as means (SD) and categorical variables as frequency (percentage)

*ASCVD * atherosclerotic cardiovascular disease, *BMI * Body mass index, *CAC*  Coronary artery calcium, *DM * Diabetes mellitus, *HDL-C * High density lipoprotein-cholesterol, MetS = metabolic syndrome, *SBP * Systolic blood pressure

The adjusted mean CAC density is shown in Table 2. Those with DM had the lowest adjusted mean CAC density and highest adjusted mean ln(Volume) and those with neither DM nor MetS had the highest CAC density with the lowest CAC volume. Association between CAC density (or CAC volume score) and disease groups was not found to be heterogenous by baseline statin treatment. Among those with DM, insulin use, or over 10 years of DM, or high 10-year ASCVD risk, or MetS were significantly associated with higher adjusted CAC volume while the CAC density was comparable (Additional file 1: Fig. S2). Among those with MetS, CAC density was associated with HbA1c while borderline associated with HDL-C and triglycerides in univariate linear regression models (Additional file 1: Table S1). After adjustment of CAC volume scores and other baseline covariates, these associations were attenuated.

**Table 2:** Adjusted CAC Volume and Density Among Those with DM or MetS

		DM (n = 668)	MetS (n = 1122)	Neither DM/MetS (n = 2028)	*p* value
CAC volume (95%CI)	Overall	79.46 (71.97–87.72)§	60.53 (55.46–66.08)‡	51.77 (48.01–55.82)	< 0.0001
	Non-statin users	72.17 (64.44–80.81)§	56.58 (51.72–61.89)*	48.61 (45.16–52.32)	< 0.0001
	Statin users	98.12 (80.60-119.44)§	69.32 (56.62–84.88)	57.24 (47.59–68.85)	< 0.0001
CAC density (95%CI)	Overall	2.54 (2.49–2.59)§	2.61 (2.57–2.65)‡	2.69 (2.65–2.72)	< 0.0001
	Non-statin users	2.56 (2.51–2.62)§	2.62 (2.57–2.66)†	2.68 (2.65–2.72)	0.0001
	Statin users	2.53 (2.44–2.62)§	2.65 (2.55–2.74)*	2.76 (2.67–2.84)	< 0.0001

The mean CAC density (or ln(Volume)) was adjusted for ln(Volume) (or CAC density), age, sex, race/ethnicity, education, smoking status, statin use and hypertension medication

*P* values were calculated from ANOVA test among three groups (last column) and from student t-tests comparing DM vs. neither DM/MetS and MetS versus neither DM/MetS (presented as symbols)

*CAC*  Coronary artery calcium, *DM*  Diabetes mellitus, *MetS*  Metabolic syndrome

*p < 0.05, †*p* < 0.01, ‡* p*< 0.001, §*p* < 0.0001.

### CAC density, CAC volume and CHD/CVD risk among DM/MetS subgroups

**Fig. 1:**
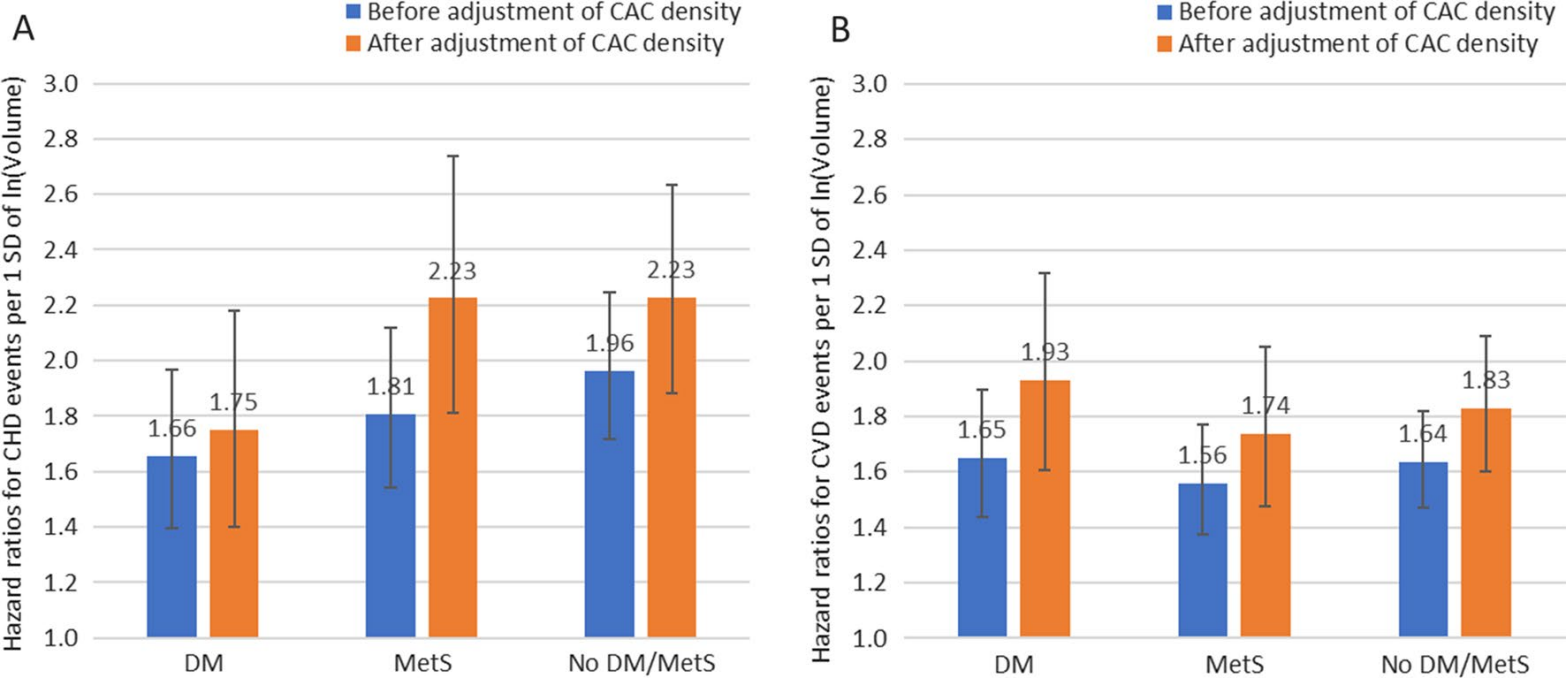
Standardized Hazard Ratios (95% Confidence Interval) of CAC Volume for CHD (**A**) and CVD (**B**) Before and After Adjustment of CAC Density by Disease Condition. Cox regression models were also adjusted for 10-year ASCVD risk, race/ethnicity, education, BMI and statin therapy. All *p* < 0.0001 for the HRs of ln(volume) score for CVD/CHD events

During a median follow-up time of 14.9 years, average CHD event rates were 21.0, 14.6 and 10.4 per 1000 person-years in those with DM, MetS and neither DM/MetS, respectively. Corresponding CVD event rates were 34.3, 23.1 and 16.7 per 1000 person-years. We compared the standardized HRs of ln(Volume) before and after adjustment of CAC density in the Cox regression model (Fig. 1). Before adjustment of CAC density, higher ln(Volume) was associated with greater CHD and CVD risks in all groups (all *p* < 0.0001). When adjusted for CAC density, the magnitude of CHD/CVD risk associated with ln(Volume) increased. The relative increase from before to after adjustment of CAC density was greatest among those with MetS for CHD events (HR changed from 1.81 to 2.23 by 23.2%) and least among those with DM for CHD events (HR changed from 1.66 to 1.75 by 5.4%).

After adjusting for ln(Volume) and other risk factors, 1 SD increase of CAC density was associated with 9%, 30% and 21% lower CHD risk and 23%, 17% and 18% lower CVD risk among those with DM, MetS and neither DM/MetS, respectively (all *p* < 0.05 except for CHD events in DM group) (Table 3). Participants with MetS in the 4th quartile of the CAC density had a 50% (*p* = 0.02) lower CHD risk and a 33% (*p* = 0.10) lower CVD risk compared to those in the 1st quartile; for those with neither DM/MetS, HRs comparing the highest vs. lowest quartile density were 0.59 for both CHD and CVD (both *p* < 0.05); for those with DM, corresponding HRs were 0.89 for CHD and 0.54 for CVD (*p* < 0.05 for CVD events). The *p* value for the multiplicative interaction tests of CAC density and disease groups were 0.84 for CVD and 0.56 for CHD; however, the point estimates of HRs for CAC density appear to vary by disease group, indicating a non-statistically significant heterogeneous association between CAC density and CHD/CVD events among those with DM or MetS. In sensitivity analysis, additional adjustment of three inflammatory biomarkers (C-reactive protein, interleukin-6, and fibrinogen) did not appreciably change the results (Additional file 1: Table S2). We additionally examined MI and CHD death as acute events and results were similar to our main analysis (Additional file 1: Table S3). Also, non-linear associations of CAC density and CHD/CVD events were examined using Spline Cox regression models (Additional file 1: Fig. S3). Compared to a CAC density of 4, the cut point of CAC density with significant risk increase (lower 95% confidence limit of HR > 1) was 2.70 for CHD and 2.73 for CVD, respectively.

**Table 3:** Hazard Ratios and 95% Confidence Interval for CHD and CVD According to Continuous CAC Density and CAC Density Quartile

	DM (n = 668)	MetS (n = 1122)	Neither DM/MetS (n = 2028)
*CHD*			
*N (%) of events*	161 (24.1%)	203 (18.1%)	276 (13.6%)
Per 1 SD of CAC density	0.91 (0.72–1.16)	0.70 (0.56–0.87)†	0.79 (0.66–0.95)*
CAC density Q2 versus Q1	0.79 (0.44–1.41)	0.88 (0.53–1.46)	0.93 (0.60–1.44)
CAC density Q3 versus Q1	0.79 (0.43–1.46)	0.67 (0.38–1.20)	0.56 (0.35–0.92)*
CAC density Q4 versus Q1	0.89 (0.47–1.67)	0.50 (0.28–0.91)*	0.59 (0.36–0.97)*
*CVD*			
*N (%) of events*	248 (37.1%)	308 (27.5%)	433 (21.4%)
Per 1 SD of CAC Density	0.77 (0.64–0.94)†	0.83 (0.70–0.99)*	0.82 (0.71–0.95)†
CAC density Q2 versus Q1	0.71 (0.45–1.10)	1.04 (0.70–1.56)	0.81 (0.58–1.12)
CAC density Q3 versus Q1	0.62 (0.38-1.00)*	1.04 (0.66–1.64)	0.53 (0.37–0.78)†
CAC density Q4 versus Q1	0.54 (0.32–0.89)*	0.67 (0.41–1.08)	0.59 (0.41–0.86)†

Models were adjusted for 10-year ASCVD risk, race/ethnicity, education, BMI, statin therapy and ln(Volume)

*ASCVD*  Atherosclerotic cardiovascular disease, *BMI*  Body mass index, *CAC* Coronary artery calcium, *CHD* Coronary heart disease, *CVD * Cardiovascular disease, *DM*  diabetes mellitus, *MetS*  metabolic syndrome

*p < 0.05, †*p* < 0.01

### Predictive value of CAC density and CAC volume scores in CHD/CVD risk assessment

Table 4 compared the Harrell’s c-statistics between the prediction model with CAC volume and CAC density (Model 4) and three other models with no CAC density (Model 1–3). The base model (10-year ASCVD risk score + race/ethnicity + BMI + statin use + education) without any CAC measures had the lowest c-statistics of 0.60–0.64 for CHD and 0.59–0.66 for CVD among the three groups. Model 4 with both CAC volume and density plus other covariates had the highest c-statistics in all three disease groups for the prediction of CHD and CVD events: the Harrell’s c-statistics were 0.67, 0.72 and 0.70 among DM, MetS, and neither DM/MetS for CHD and 0.67, 0.70 and 0.71 for CVD, respectively. Models with either Agatston score only (Model 2) or CAC volume only (Model 3) showed slightly poorer discrimination ability than Model 4 however comparisons to Model 4 were mostly not statistically significant, except for CHD events among those with MetS. In addition, we examined a 5th model of base model + ln(Agatston score) + CAC density: the Harrell’s c-statistics were identical to the 2 decimal place as Model 4 (base model + ln(Volume) + CAC density). In sensitivity analysis, including CAC density as quartile variables did not appreciably change the c-statistics.

**Table 4:** Comparison of C-statistics for Discriminating CHD and CVD Risks Between Models with and Without CAC Density

	DM (n = 668)	MetS (n = 1122)	Neither DM/MetS (n = 2028)	Total (n = 3818)
*CHD*				
Model 1	0.60†	0.64§	0.63‡	0.63§
Model 2	0.67	0.70†	0.70	0.69*
Model 3	0.67	0.70*	0.70	0.70
Model 4	0.67	0.72	0.70	0.70
*CVD*				
Model 1	0.59‡	0.65†	0.66§	0.66§
Model 2	0.66	0.69	0.70	0.69*
Model 3	0.66	0.69	0.70	0.69
Model 4	0.67	0.70	0.71	0.70

Model 1 (Base model): 10-year ASCVD risk score + race/ethnicity + education + BMI + statin therapy

Model 2: Base model + ln(Agatston)

Model 3: Base model + ln(Volume)

Model 4: Base model + ln(Volume) + CAC Density

Harrell’s c-statistics were compared between model 4 versus model 1, 2 or 3

*ASCVD*  Atherosclerotic cardiovascular disease, *BMI*   Body mass index, *CAC*  Coronary artery calcium, *CHD*   Coronary heart disease, *CVD * Cardiovascular disease, *DM*  Diabetes mellitus, *MetS* , Metabolic syndrome

*p < 0.05, †*p* < 0.01, ‡*p* < 0.001, § *p* < 0.0001

## Discussion

Our study showed CAC density to be lower in persons with DM or MetS and CAC density to be inversely related to future CVD and CHD risk in subjects with MetS or neither DM/MetS, and for CVD in those with DM. In addition, only in those with MetS did CAC density show incremental predictive value for CHD when added to models with traditional risk factors and CAC volume score. Current clinical practice focuses on use of the Agatston CAC score. We have shown CHD and CVD risk can be further modified by CAC density, thus CHD or CVD risk based on the Agatston score alone may be underestimated when CAC density is low. Importantly, among all 3 patient groups, risks associated with higher CAC volume increased after adjustment for CAC density, suggesting risk associated with CAC volume could be potentially masked if not taking CAC density into account.

The density of coronary plaque may be influenced by other CVD risk factors. Kwan et al. found that obesity was associated with non-calcified plaque [17]. Previous findings showed that persons with DM or MetS have a higher prevalence of non-calcified plaque with lipid-rich components being more responsible for acute coronary syndrome [18–20]. Moreover, patients with MetS are also found to have more severe coronary artery stenosis which is less related to heavily calcified plaques compared to non-MetS individuals [21]. When calcified plaque gets denser, it may represent a more “mature” plaque, which is considered to be more stable and less prone to plaque rupture and, consequently, fewer incident CHD events.

Association of CAC density and CVD seems stronger among those with DM, which could be related to either the increased event number and power for CVD events or because the inclusion of HF as important CVD manifestation for those with DM. In another MESA study, it was found that CAC density had inverse association with CVD events similarly in DM and non-DM [22], while another study found the CAC density was positively associated with higher CVD mortality just like other CAC measures among patients with DM [23].

The 2018 AHA/ACC lipid management guidelines recommend CAC scanning as supplementary ASCVD risk assessment tools when preventive strategies are unclear [24]. Yet the current algorithm of CAC quantification has faced several challenges, including the underlying assumption that denser plaque is related to higher CVD risk [25]. However prior population and biomedical studies found large, dense plaques are indicators of stable, healed plaques with less tendency to rupture and cause vessel obstruction [4, 5, 9, 26, 27]. On the contrary, microcalcification, which is hard to detect by cardiac CT scanning predicts acute coronary events and in an intravascular ultrasound study was shown to be associated with accelerated disease progression compared to macrocalcification. Pugliese et al. [28] noted molecular mechanisms regulating transition from “microcalcification” to “macrocalcification” were anti-inflammatory therefore stabilizing vascular plaque [27].

In the current study we found that CAC density was lower among those with DM and MetS compared to those without DM/MetS while CAC volume score was higher. This is consistent with a prior MESA study finding CAC density to be inversely associated with DM [29]. Among the subjects with detectable CAC, DM and other cardiometabolic risk factors may induce inflammation preventing the normal calcification of plaque and instead induce spotty calcification and larger but more lipid-rich plaque [27]. Moreover, statin use has been shown to be associated with CAC progression by reduced lipid core and increases in CAC density [30–32]. We found statin users had significantly higher CAC volume in all three disease groups, while association between CAC density and DM/MetS was not found to be influenced by baseline statin use. It should be noted that we examined baseline mean CAC density and baseline statin use, whose cross-sectional association may not necessarily reflect the effect of statins on density given prior statin dose and duration were unknown. In addition, the baseline of the current study was 2000–2002 for most included subjects, which may explain the relative low statin use in DM and MetS population. This could have also modulated the lack of association which was observed between statin usage and plaque density. Finally, some of the included subjects had newly found calcification from MESA exam 2 and 3 whose density measure was not largely influenced by statin use.

Our study has strengths and limitations. An important strength is that MESA has standardized assessment of risk factors, coronary calcium, and adjudication of CHD and CVD events across the field sites, as well as representation of four major US ethnic groups. However, our analyses were limited to the MESA sample with only non-zero Agatston scores (for which CAC density was quantifiable); thus, our reported event rates are likely to be higher than those of other studies in persons with MetS or DM either with or without CAC. It was previously reported that non-calcified plaque exists in those without CAC and those presenting with acute coronary events have a higher burden of non-calcified plaque than calcified plaque [33]. CT angiography may be used to detect non-calcified plaque in such case to help evaluate associated CVD risk. In addition, since the density is the average of density units on CT slides, it has a peak of score 4 for those with extremely high Hounsfield Unit (HU) plaques, thereby resulting in a “ceiling effect” where those significantly denser are weighted the same as those just meeting the threshold of 400 HU. A variable HU attenuation could potentially lead to similar density scores which may further inject variability in the outcome relationships as seen in the current study. A more accurate calcium content in each plaque can be estimated by using the average density of the entire pixels of each single plaque as a continuous HU unit (not measured in current MESA study). Such density definition has previously yielded better reproducibility and has the potential to improve the density contribution for the events prediction in future studies [34].

## Conclusion

To conclude, we showed CAC density to be lower in persons with MetS or DM compared to those with neither condition. CAC density was inversely related with future CHD and CVD events in those with DM or MetS. Including density in the prediction models for CHD, particular in those with MetS may strengthen the predictive ability of CAC volume score and improve overall risk prediction in these populations. Future investigation should confirm the predictive value of CAC density in larger DM and MetS populations before a change in diagnostic or therapeutic practice.

## Supplementary Information

**Additional file 1**. Supplementary materials.

## Data Availability

The data that support the findings of this study are available from MESA but restrictions apply to the availability of these data. Data are however available from the authors upon reasonable request and with permission of Publication and Presentation Committee of MESA. The details to access MESA data can be found on MESA official website.

## Abbreviations

ASCVD: Atherosclerotic cardiovascular disease
BMI: Body mass index
CAC: Coronary artery calcium
CHD: Coronary heart disease
CVD: Cardiovascular disease
DM: Diabetes mellitus
HDL-C: High density lipoprotein-cholesterol
HF: Heart failure
MESA: Multiethnic Study of Atherosclerosis
MetS: Metabolic syndrome
MI: Myocardial infarction
SBP: Systolic blood pressure
