# Supplementary material for: Coronary calcium density in relation to coronary heart disease and cardiovascular disease in adults with diabetes or metabolic syndrome: the Multi-ethnic Study of Atherosclerosis (MESA)
Source: BMC Cardiovasc Disord. 2022 Dec 9;22:536. doi: 10.1186/s12872-022-02956-4 (PMC9733236; doi:10.1186/s12872-022-02956-4)

| Supplementary Table 1. Unadjusted and adjusted association of CAC density and HbA1c and blood lipid measures among those with MetS | | |
| --- | --- | --- |
|  | Unadjusted beta coefficient | Adjusted beta coefficient |
| HbA1c, per 1 SD | 0.146 (0.060 - 0.231)^†^ | 0.021 (-0.042 - 0.084) |
| HDL-C, per 1 SD | -0.059 (-0.119 - 0.001)* | 0.012 (-0.037 - 0.061) |
| LDL-C, per 1 SD | 0.002 (-0.042 - 0.045) | -0.002 (-0.034 - 0.031) |
| Total cholesterol, per 1 SD | -0.004 (-0.046 - 0.038) | 0.005 (-0.026 - 0.037) |
| ln(Triglycerides), per 1 SD | 0.022 (-0.028 - 0.072)* | 0.001 (-0.038 - 0.040) |
| Multivariable models were adjusted for age, sex, race/ethnicity, education, statin therapy, hypertension medication and ln(volume).  Triglycerides were log transformed to get normal distribution.  1 SD of HbA1c = 1.04 %; 1 SD of HDL-C = 14.39 mg/dL; 1 SD of LDL-C = 32.12 mg/dL; 1 SD of total cholesterol = 36.86 mg/dL; 1 SD of ln(triglycerides) = 0.53 mg/dL.  *p < 0.1, ^†^ p < 0.001.  Abbreviations: CAC = coronary artery calcium; HbA1c = hemoglobin A1c; HDL-C = high density lipoprotein-cholesterol; LDL-C = low density lipoprotein-cholesterol; MetS = metabolic syndrome. | | |

| Supplementary Table 2. Hazard ratios and 95% CI for CHD and CVD according to continuous density and density quartiles additionally adjusted for inflammatory biomarkers. | | | |
| --- | --- | --- | --- |
|  | DM  (n = 554) | MetS  (n = 916) | Neither DM/MetS  (n = 1,779) |
| CHD | | | |
| Per 1 SD of Density | 0.86 (0.66-1.11) | 0.67 (0.52-0.85)^‡^ | 0.77 (0.64-0.94)* |
| CAC Density Q2 vs. Q1 | 0.68 (0.37-1.26) | 0.77 (0.44-1.33) | 0.76 (0.47-1.22) |
| CAC Density Q3 vs. Q1 | 0.67 (0.36-1.28) | 0.56 (0.31-1.04) | 0.49 (0.29-0.83)^†^ |
| CAC Density Q4 vs. Q1 | 0.77 (0.40-1.50) | 0.44 (0.23-0.83)* | 0.52 (0.31-0.87)* |
|  |  |  |  |
| CVD | | | |
| Per 1 SD of Density | 0.75 (0.61-0.93)^†^ | 0.81 (0.67-0.98)* | 0.80 (0.69-0.94)^†^ |
| CAC Density Q2 vs. Q1 | 0.70 (0.43-1.14) | 0.91 (0.57-1.44) | 0.72 (0.50-1.03) |
| CAC Density Q3 vs. Q1 | 0.56 (0.33-0.94)* | 0.90 (0.55-1.50) | 0.51 (0.34-0.76)^‡^ |
| CAC Density Q4 vs. Q1 | 0.52 (0.30-0.89)* | 0.61 (0.36-1.04) | 0.55 (0.37-0.82)^†^ |
| Models were adjusted for 10-year ASCVD risk, race/ethnicity, education, BMI, statin therapy, ln(volume), hs-CRP, IL-6 and fibrinogen.  Hs-CRP, IL-6 and fibrinogen were only tested in Exam 1, the above analysis was limited to those with non-zero Agatston score in Exam 1.  *p < 0.05, ^†^ p < 0.01, ^‡^p < 0.001.  Abbreviations: ASCVD = atherosclerotic cardiovascular disease; BMI = body mass index; CAC = coronary artery calcium; CHD = coronary heart disease; CVD = cardiovascular disease; DM = diabetes mellitus; hs-CRP= high sensitivity C-reactive protein; IL-6 = interleukin 6; MetS = metabolic syndrome. | | | |

| Supplementary Table 3. Hazard ratios and 95% CI for MI and CHD death according to continuous density and density quartiles. | | | |
| --- | --- | --- | --- |
|  | DM  (n = 668) | MetS  (n = 1,122) | Neither DM/MetS  (n = 2,028) |
| MI | | | |
| Per 1 SD of Density | 0.77 (0.53-1.13) | 0.72 (0.52-1.01) | 0.73 (0.54-0.98)* |
| CAC Density Q2 vs. Q1 | 0.63 (0.25-1.58) | 0.79 (0.37-1.65) | 0.88 (0.43-1.80) |
| CAC Density Q3 vs. Q1 | 0.64 (0.25-1.66) | 0.61 (0.26-1.43) | 0.66 (0.30-1.44) |
| CAC Density Q4 vs. Q1 | 0.44 (0.16-1.21) | 0.46 (0.19-1.12) | 0.49 (0.22-1.09) |
|  |  |  |  |
| CHD death | | | |
| Per 1 SD of Density | 0.84 (0.53-1.33) | 0.56 (0.33-0.95)* | 0.96 (0.65-1.42) |
| CAC Density Q2 vs. Q1 | 1.40 (0.49-3.97) | 0.49 (0.16-1.50) | 1.29 (0.46-3.57) |
| CAC Density Q3 vs. Q1 | 1.13 (0.35-3.65) | 0.51 (0.15-1.74) | 1.05 (0.35-3.14) |
| CAC Density Q4 vs. Q1 | 1.17 (0.34-4.05) | 0.34 (0.09-1.26) | 1.09 (0.36-3.31) |
| Models were adjusted for 10-year ASCVD risk, race/ethnicity, education, BMI, statin therapy and ln(volume).  *p < 0.05.  Abbreviations: ASCVD = atherosclerotic cardiovascular disease; BMI = body mass index; CAC = coronary artery calcium; CHD = coronary heart disease; DM = diabetes mellitus; MI = myocardial infarction; MetS = metabolic syndrome. | | | |

Supplementary figures.

Supplementary Figure 1. Study Design


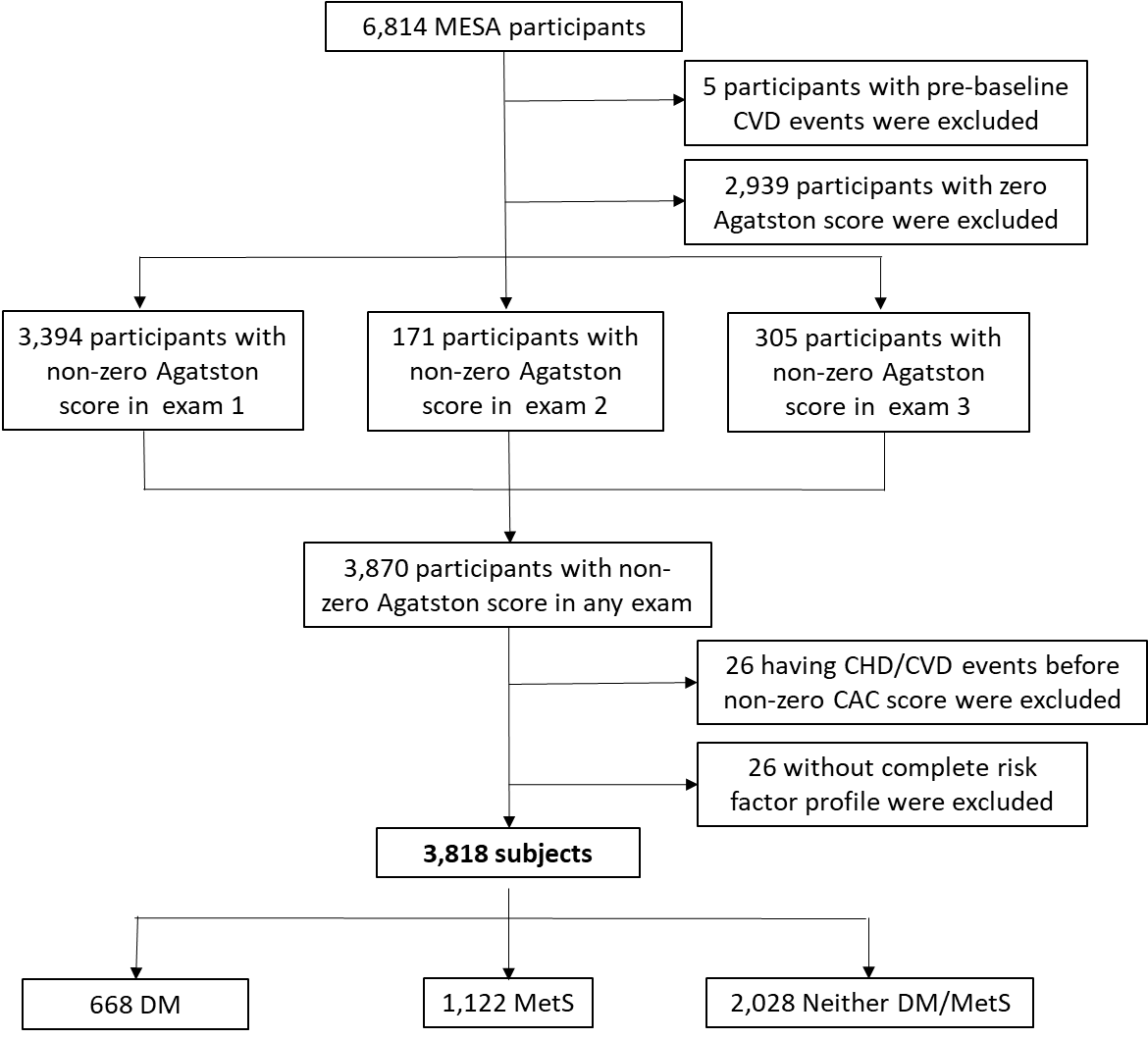


Supplementary Figure 2. Adjusted CAC volume score and CAC density by DM severity


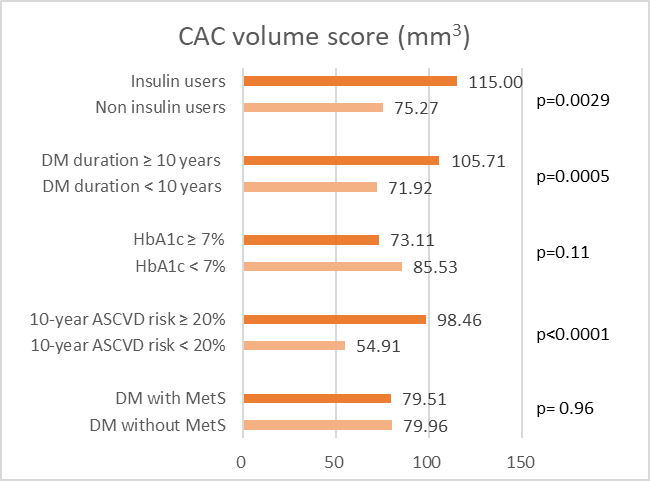


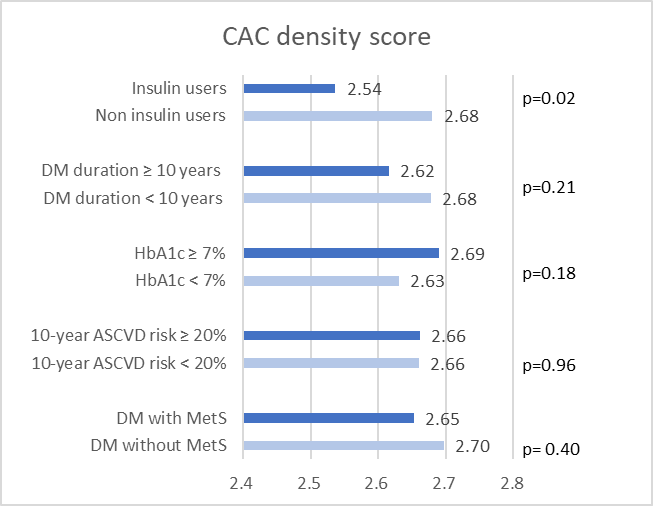


Supplementary Figure 3. Non-linear association of CHD/CVD risk and CAC density in spline Cox PH regression model.


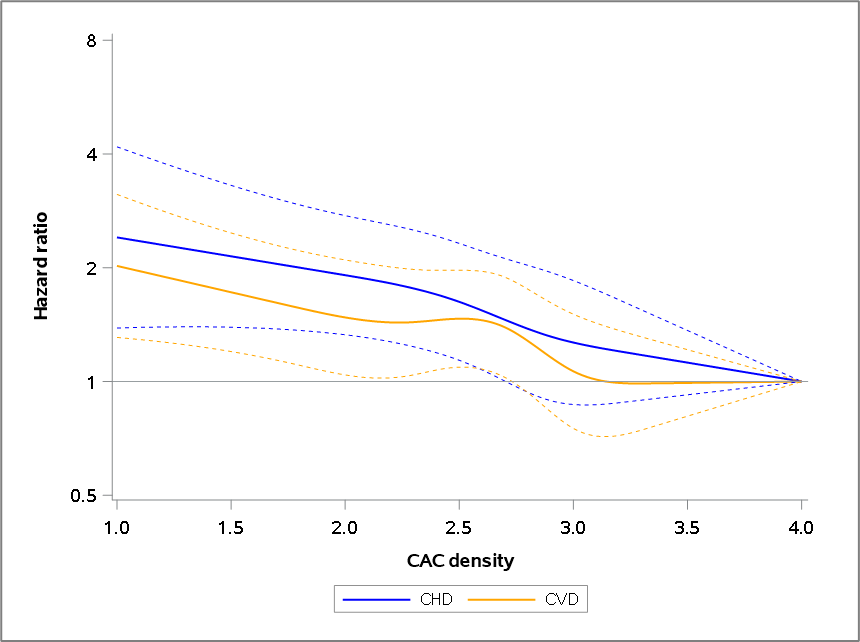

Supplement: Supplementary file 1 — Additional file 1. Supplementary materials. [file 12872_2022_2956_MOESM1_ESM.docx]
